# *In Vitro* Mechanical Properties of Mineral Trioxide Aggregate in Moist and Dry Intracanal Environments 

**DOI:** 10.22037/iej.v13i1.15561

**Published:** 2018

**Authors:** Radovan Žižka, Radim Čtvrtlík, Jan Tomaštík, Kamila Fačevicová, Ladislav Gregor, Jiří Šedý

**Affiliations:** a *Institute of Dentistry and Oral Sciences, Faculty of Medicine and Dentistry, Palacky University, Olomouc, Czech Republic; *; b *Czech Educational and Dental Research Innovative Group (CEDRIG), Brno, Czech Republic;*; c *Joint Laboratory of Optics, Palacky University and Institute of Physics, Academy of Sciences of the Czech Republic, Olomouc, Czech Republic; *; d *Department of Mathematical Analysis and Applications of Mathematics, Faculty of Science, Palacky University, Olomouc, Czech Republic; *; e *Department of Anatomy, Faculty of Medicine and Dentistry, Palacky University, Olomouc, Czech Republic*

**Keywords:** Microhardness, Mineral Trioxide Aggregate, Modulus of Elasticity, Nanoindentation

## Abstract

**Introduction:**

The purpose of this study was to examine the microhardness and modulus of elasticity (MOE) of White ProRoot MTA (Dentsply Tulsa Dental, Tulsa, OK) after setting in moist or dry intracanal conditions.

**Methods and Materials:**

To simulate root canal system, 14 polyethylen molds with internal diameter of 1 mm and height of 12 mm were used. These molds were filled with 9-mm thick layers of White ProRoot Mineral Trioxide Aggregate (MTA; Dentsply Tulsa Dental, Tulsa, OK). The experimental group (*n*=7) had a damp cotton pellet with 1.5 mm height and a 1.5 mm layer of resin composite placed on it. In control group (*n*=7) the whole 3 mm above MTA were filled with resin composite. The specimens were kept in 37^°^C and relative humidity of 80% for 4 days in order to simulate physiological conditions. Specimens were longitudinally sectioned and nanoindentation tests were carried out using Berkovich indenter at loading rate of 2 mN/s at 4×5 matrices of indents which were located in the coronal, middle and apical thirds of the specimen’s cross section, to evaluate the microhardness and modulus of elasticity of the specimen to appraise the progression of the setting process. Differences were assessed using nonparametric generalized Friedman rank sum and Wilcoxon Rank-Sum tests.

**Results:**

Statistical analysis showed that there was a significant difference in microhardness and MOE between control and experimental groups at coronal (*P*<0.001), middle (*P*<0.001) and apical (*P*<0.001) thirds of the simulated rod from simulated apical foramen. Kruskal-Wallis test showed no significant effect of depth on microhardness of material in experimental or control groups.

**Conclusion:**

Within limitations of this *in vitro* study, it seems that moist intracanal environment improves setting of MTA in various depths.

## Introduction

Mineral Trioxide Aggregate (MTA) has become an essential tool for endodontists and general dental practitioners. It was first introduced as root-end filling as well as perforation repair material because of its excellent biocompatibility and possible acceptance of moisture during setting reaction [1]. Moreover, it is being widely used for direct pulp capping, root perforation repair, pulpotomy, apexification, regenerative endodontic procedures [2-4] and complete root canal obturation [5, 6]. 

According to the manufacturer´s instructions, MTA as a hydraulic cement which sets in moist condition at least for 4 h. Wet cotton pellet placed against the coronal surface of MTA is vastly used for this purpose. In literature, the use of wet cotton pellet for each indication of MTA is recommended [2], but some evidence indicate that it´s use is not beneficial if thickness of MTA is less than 4 mm [7], or if the apical foramen is larger than 1 mm [8]. If wet cotton pellet is used, a second appointment is necessary in order to remove the cotton pellet and place a final restoration. This approach has cost and convenience implications as well as the risk of reinfection of the root canal system [8, 9].

**Figure 1:**
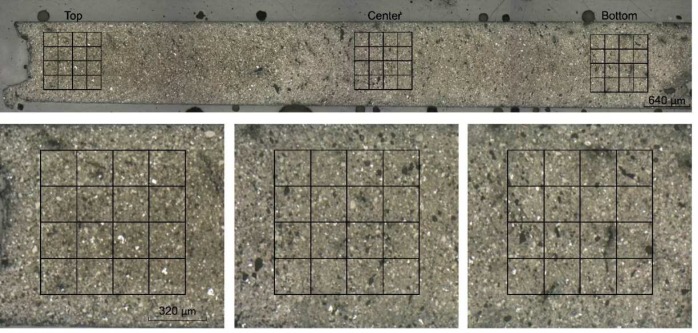
Specimen and indent´s matrices (image taken with confocal microscope)

Investigation of the mechanical properties of contemporary dental materials at relevant scale is necessary for understanding their performance, application limits and of course, for optimization of their application protocols. It should be noted that mechanical properties show an important size-dependent character [10]. One of the mostly mentioned mechanical properties of MTA related to setting is microhardness. It is a crucial parameter characterizing its mechanical strength and the quality of setting as well.

The purpose of this study was to examine the role of intracanal humidity on microhardness and elastic modulus of MTA after setting in simulated root canal environment and if it is possible to use elastic modulus as an indicator of setting MTA. This has been measured using nanoindentation test with MTA exposed to moist and dry intra-canal environment.

## Materials and Methods

In this *in vitro *experimental study, polyethylen mold tubes with internal diameter of 1 mm and height of 12 mm were used to simulate the root canal environment. The molds were randomly divided to 2 groups (experimental and control), with 8 molds in each group. 

In the experimental group, the end of tube was sealed with 1.5 mm thick layer of flowable resin composite and after light curing, a 1.5 mm thick layer of wet cotton was placed inside in order to simulate wet intracanal environment. In control group, the end of mold tube was sealed with 3 mm thick layer of flowable resin composite, without any intracanal humidity. In both groups, the tooth-colored ProRoot MTA (Dentsply, Tulsa Dental, Tulsa, OK, USA) was mixed with sterile distilled water according to the manufacturer’s instructions and incrementally delivered and vertically compacted with #3 Machtou plugger. After this procedure, a 9 mm long cylinders of material in molds were obtained. 

Subsequently whole external surface of mold except simulated foramen was covered with nail polish (Nail lacquer, O.P.I, North Holywood, CA, USA) in order to avoid absorption of moisture apart from simulated apical foramen [11]. The specimens were kept in 37^°^C and relative humidity of 80% for 4 days in order to simulate physiological conditions.

After that the cylindrical samples were fixed in to the acrylic resin Dentacryl (SpofaDental, Jičín, Czech Republic) and were sectioned. Subsequently were specimen grid by sequential procedure with 600 and 800-grit silicon carbide and subsequently polished using 0.25 µm diamond suspension. All the preparation procedures were performed under the conditions of water cooling in order to prevent the sample from overheating. At the end of this procedure, the cross-sectional surface of dimensions 1 mm×9 mm with the sufficiently low surface roughness was acquired.

Nanoindentation experiments were carried out using a fully calibrated NanoTest instrument (Micro Materials, Wrexham, UK) in load controlled mode at room temperature. During the test the normal load of 40 mN was applied to the diamond Berkovich indenter (Synton-MDP, Port, Switzerland) at loading rate of 2 mN/s. The corresponding penetration depths were typically around 1 µm. In order to obtain the complete picture of the effect of the moisture on the mechanical properties of MTA the 4×5 matrices of indents were located in the central and opposite ends of the specimen’s cross section (Figure 1). The distance of the indents in the matrix was 200 µm. Indentation hardness and reduced modulus of elasticity (MOE) were calculated using the standard procedure based on the analysis of the load-displacement record [12].

**Figure 2:**
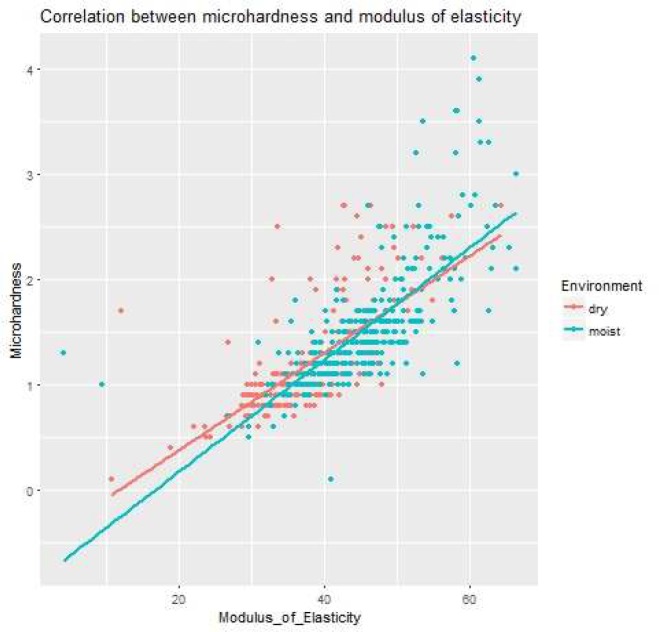
Scatter plot of microhardness and modulus elasticity of MTA

***Statistical analysis***

Due to non-normal distribution of hardness and modulus of elasticity, the effect of depth of material and presence of dry or moist intracanal environment were analysed with generalized Friedman rank sum test and Wilcoxon Rank-Sum test. Statistical significance was set at 0.05.

## Results

The descriptive statistics of the data are summarized in table 1. The data do not exhibit normal distribution according to the Shapiro-Wilk tests. Statistical analysis employing generalized Friedman rank sum test for blocked data showed that there was no significant difference in surface microhardness in various depths of material for dry (*P*=0.1061) or moist intracanal environment (*P*=0.0962). 

The influence of dry and wet environment on hardness and MOE in stated depth was tested by Wilcoxon Rank-Sum test. The results indicated that there was significant difference in microhardness and MOE at first, second and last third of material from simulated apical foramen, when higher values of observed variables were typical for wet environment and all the obtained *P*-values were substantially lower than significance level 0.05. Moreover, there was quite strong correlation between microhardness and MOE; the Spearman correlation coefficients in dry and moist intracanal environment were 0.77 and 0.78, respectively. Its zero values were rejected by the Spearman’s tests (both *P*<0.05). The linear relationship is also visible from the scatter plot of microhardness and MOE (Tables 1 and 2) (Figure 2). 

## Discussion

The present *in vitro* study compare the microhardness and MOE of MTA in dry and wet environments. Microhardness testing is based on the evaluation of resistance of material againstplastic/elastic deformation [13]. This resistance is influenced by many material properties such as MOE, Poisson’s ratio, yield strength, stress hardening exponent, tensile strength and many others [14, 15] as well as the experimental conditions. Conventionally, the hardness test is based on the measurement of the diagonal dimensions of the residual plastic impression created during the indentation process, where the hard indenter is pressed into the sample’s surface. Vickers indenter, a four-sided pyramid, was used in most studies. This approach relies on the visual observation of residual indentation, and also on the assumption that only very limited elastic recovery of the residual indent takes place. Generally, the measurement of the actual size of the indent becomes intricate with the decrease of its dimensions and increase of surface roughness. On the contrary, the advanced approach of hardness testing, also called depth sensing indentation, is based on the continuous recording of the force-displacement data during the whole test. Such approach includes loading, creep and unloading. The graphical representation of such data is called a nanoindentation curve, containing the loading and unloading curves. The defined geometry of the indenter together with the measured penetration depth are then used for determination of the projected area and in turn for calculation of hardness. This reduces the possible errors of the visual observation of the residual impression. The obvious advantage of depth sensing indentation is the ability to determine the elastic modulus of the specimen evaluating the slope of the unloading branch [12]. Also, the energy of elastic and plastic works associated with the indentation process can also be calculated, which makes the analysis far more comprehensive [16, 17]. Finally, other phenomena like creep, incipient plasticity [18] or other pressure-induced phase transformations [19] can be studied, even at elevated temperatures [16]. 

**Table 1:** Descriptive statistics of measured data of microhardness

**Depth (N)**	**Mean (SD) in moist condition**	**Depth (N)**	**Mean (SD) in dry condition**
**Coronal (95)**	1.52 (0.46)	**Coronal (78)**	1.14 (0.40)
**Middle (117)**	1.42 (0.36)	**Middle (85)**	1.30 (0.52)
**Apical (101)**	1.40 (0.33)	**Apical (82)**	1.27 (0.56)

**Table 2:** Descriptive statistics of measured data of elastic modulus

**Depth (N)**	**Mean (SD) in moist condition**	**Depth (N)**	**Mean (SD) in dry condition**
**Coronal (110)**	46.94 (8.02)	**Coronal (84)**	36.72 (7.28)
**Middle (127)**	45.60 (7.35)	**Middle (87)**	40.56 (8.74)
**Apical (107)**	42.75 (7.93)	**Apical (85)**	37.72 (7.82)

In recent studies, specimens were subjected to Vickers microhardness test only in horizontal plane and in specific distance from simulated apical foramen. Several studies suggest that dry or moist intracanal environment does not influence the setting of MTA up to 2 mm [20] or 4 mm [7, 8]. Microhardness tests have been used for the evaluation of the quality and progression of the hydration process and as an indicator of the setting process [21, 22]. Previous work marked on its capability to provide information on the effect of setting conditions and the strength of tested materials [23]. For accurate comparison with other materials, specimens should be polished and dimensions of at least 6 mm thick and 12 mm wide are required. These conditions are far from clinical endodontics. Thus, tests in this field are mainly comparative for the use within each study [24]. Unlike all previous studies, we measured microhardness of real size samples sectioned to longitudinal axis. The loading force was chosen in order to keep the local characteristics of the measurements on one hand and to avoid the negative effect of surface roughness on the other hand. 

To our knowledge, in this study, the mechanical properties (indentation hardness and elastic modulus) of MTA using nanoindentation were studied for the first time. Furthermore, the cross-sectioned area was tested in order to get the complete hardness map. In addition, the nanoindentation depth sensing approach allowed us to explore reduced modulus distribution also. Since all the tests were performed under the same conditions, both the relative as well as absolute values of mechanical properties across the longitudinal cross section were provided. 

In general, the data demonstrated high standard deviations ranging from 21% to 45% for microhardness and from 11% to 19% for elastic modulus. The large variability in standard deviations may have resulted from local differences in MTA itself or caused by compaction of material. The maintenance of a constant degree of hydration of MTA during compaction is almost impossible in clinical setting [8]. Another possible cause may be continually maturing nature of material because of continuation of hydration reactions and structure maturation in MTA, well beyond clinically observed setting times [25]. High standard deviations can be found in other studies dealing with microhardness of MTA as well [7, 8].

Our findings suggest that additional water from wet intracanal environment can improve the setting of white MTA along whole thickness of material. Nevertheless, *in vivo*, there are another possible sources of moisture such as absorption through root [11] which can improve setting of white MTA. However, this study aimed to investigate and to compare the microhardness and MOE only in relation to intracanal environment. Although clinical impact of less set material in root canal when used as root canal filling is questionable. Some evidence suggests that the apical seal can be obtained independently on wet cotton pellet placement [26] and rather is connected to thickness of MTA plug [27]. We must emphasize that the outstanding sealing properties of MTA are most probably caused by slight setting expansion and production of interfacial layer [28, 29] which are dependent on the setting of material.

## Conclusion

Within limitations of this study, it seems that the use of wet cotton pellet is beneficial for the whole thickness of material when MTA used as a root canal filling material. Moreover, the microhardness and elastic modulus were significantly higher even for apical third. It seems that usage of wet cotton pellet even in situations where only limited thickness of MTA is used like revascularization/revitalization or perforation repair, is not redundant. Because of *in vitro* nature of this experiment, the authors suggest caution when extrapolating the results to clinical practice.
